# A comprehensive DNA panel next generation sequencing approach supporting diagnostics and therapy prediction in neurooncology

**DOI:** 10.1186/s40478-020-01000-w

**Published:** 2020-08-05

**Authors:** Julia Lorenz, Tanja Rothhammer-Hampl, Saida Zoubaa, Elisabeth Bumes, Tobias Pukrop, Oliver Kölbl, Selim Corbacioglu, Nils O. Schmidt, Martin Proescholdt, Peter Hau, Markus J. Riemenschneider

**Affiliations:** 1 Department of Neuropathology, Regensburg University Hospital, Franz-Josef-Strauss-Allee 11, 93053 Regensburg, Germany; 2 Wilhelm Sander Neuro-Oncology Unit, Regensburg University Hospital, Regensburg, Germany; 3 Department of Neurology, Regensburg University Hospital, Regensburg, Germany; 4 Department of Internal Medicine III, Regensburg University Hospital, Regensburg, Germany; 5 Department of Radiotherapy, Regensburg University Hospital, Regensburg, Germany; 6 Department of Pediatric Hematology, Oncology and Stem Cell Transplantation, Regensburg University Hospital, Regensburg, Germany; 7 Department of Neurosurgery, University Hospital Regensburg, Regensburg, Germany

**Keywords:** Glioblastoma, Glioma, Meningioma, Medulloblastoma, Next generation sequencing, Targeted therapy, Integrated diagnoses

## Abstract

Recent updates in the classification of central nervous system (CNS) tumors have increased the need for molecular testing. Assessment of multiple alterations in parallel, complex combinations of gene sequence and chromosomal changes, as well as therapy prediction by identification of actionable mutations are the major challenges. We here report on a customized next generation sequencing (NGS)-based DNA panel assay that combines diagnostic and predictive testing and -as a comprehensive approach- allows for simultaneous single nucleotide variant (SNP) / small insertion/deletion (InDel), copy number variation (CNV) and loss of heterozygosity (LOH) detection. We analyzed formalin-fixed and paraffin-embedded (FFPE) DNA from a total of 104 patients with CNS tumors. After amplicon capture-based library preparation, sequencing was performed on the relatively cost-efficient Illiumina MiniSeq platform and evaluated with freely available bioinformatical tools. 57 genes for exonic SNP/InDel calling (19 of those in intronic regions for CNV analysis), 3 chromosomal arms and 4 entire chromosomes for CNV and LOH analysis were covered. Results were extensively validated. Our approach yielded high accuracy, sensitivity and specificity. It led to refined diagnoses in a relevant number of analyzed cases, reliably enabled complex subclassifications (e.g. for medulloblastomas) and identified actionable targets for clinical use. Thus, our single-platform approach is an efficient and powerful tool to comprehensively support molecular testing in neurooncology. Future functionality is guaranteed as novel upcoming biomarkers can be easily incorporated in a modular panel design.

## Introduction

Diagnostic neurooncology has to deal with an increasing number of molecular markers for classification and therapy prediction. With the 2016 WHO classification of central nervous system (CNS) tumors, testing for molecular alterations is mandatory for some entities [1]. For example, the *IDH* mutational status and codeletion of the chromosomal arms 1p/19q need to be determined for the integrated diagnosis of an oligodendroglioma, *IDH*-mutant and 1p/19q-codeleted. This has to be regarded as a continuous development as beyond the current classification several additional markers are being proposed that will likely be integrated in upcoming revisions of the classification. The cIMPACT-NOW consortium, e.g., recently discussed the provisional entity of *IDH*-wildtype astrocytomas in terms of their heterogeneous outcomes. They recommended *TERT* promotor (*TERTp*) mutation, *EGFR* highcopy amplification and/or combined chromosome 7 gain and 10 loss as a marker to identify tumors with glioblastoma-like outcome on a molecular scale [2]. Also, for the group of *IDH*-mutant astrocytomas a reworked grading system was proposed, acknowledging homozygous loss of *CDKN2A* as a main factor of unfavorable prognosis [3].

WHO grading and classification of meningiomas is still mainly based on histological characteristics [1]. Tumor behavior and risk of recurrence for each individual patient is often difficult to predict, even when main risk factors like patients’ age, tumor size and extent of resection, as expressed by the Simpson grade scale, are considered [4, 5]. Several recent publications try to mitigate this uncertainty by defining molecular biomarkers or subgroups of tumors with a more favorable prognosis, reviewed in 2019 from the International Consortium on Meningiomas [5]. Beside the methylation-based subgrouping approach [6], there are several prognostic molecular biomarkers that appear meaningful in meningiomas. Unfavorable variations are *CDKN2A* loss, 1p loss and *TERTp* mutations [1, 6], while *TRAF7*, *KLF4*, *AKT1* and *SMO* mutations are associated with a rather favorable prognosis [7]. It has been further described for meningioma that an increase in the complexity of copy number variations (CNV) is correlated with a higher WHO grade [4, 5].

For medulloblastoma there is a more far-reaching consensus on molecular subgrouping. Since 2012 medulloblastoma can be divided into 4 different subgroups based on transcriptomics [8]. In the 2016 WHO classification the subgrouping proposal of the initial publication was partly considered and the following 4 subgroups were defined: WNT-activated, SHH-activated/*TP53*-wildtype, SHH-activated/*TP53*-mutant and non-WNT/non-SHH-activated [1]. Each of the 4 subgroups shows specific variations [9]. In the following, also immunohistochemistry-based approaches have been suggested to identify the 4 subgroups by using a set of 5 antibodies [10].

Covering this increasing amount of molecular markers with single gene assay approaches is often not expedient. Particularly in case multiple alterations on the gene and cytogenetic level have to be assessed in parallel, like e.g. for medulloblastomas, the application of high-throughput approaches appears indispensable. Combinations of i) methylation arrays for aspects connected to tumor classification [6, 11, 12] and ii) targeted next generation sequencing (NGS) for the identification of actionable mutations [13] are a suitable approach to address this problem. However, high initial investment and annual costs for running two platforms in parallel hinder a fast expansion of the new techniques in the breadth of neuropathology. As a possible alternative, we aimed to develop a comprehensive NGS-based approach that should meet the following prerequisites: 1.) Coverage of all diagnostically relevant molecular alterations in astrocytic and oligodendroglial tumors, meningiomas and medulloblastomas for integrated diagnoses according to the 2016 WHO classification and beyond, 2.) cross-entity coverage of actionable mutations and alterations associated with therapy resistance. In order to fulfil these requirements the panel had to be designed in a way to reliably enable single nucleotide variant (SNP) and small insertion/deletion (InDel) calling as well as CNV and loss of heterozygosity (LOH) analysis. For LOH analyzes and to filter somatic aberrations from germline variations a matching blood sample had to be requested form each patient. The workflow had to be suitable for the framework of a quality-controlled diagnostic lab, i.e. in-house sequencing facility with optimal cost efficiency and timely turnaround time as well as suitability for formaldehyde-fixed and paraffin-embedded (FFPE) archival tissue samples.

## Materials and methods

### Tissue samples

In total, FFPE tissues from 104 patients with CNS tumors were analyzed. Samples were collected from the Neuropathology Department of Regensburg University Hospital (Regensburg, Germany) in line with local ethics board approval. All tumors were classified according the WHO 2016 diagnostic criteria [1]. In detail, 19 astrocytic gliomas, *IDH*-mutant (WHO grade II/III/IV); 14 oligodendrogliomas, *IDH*-mutant and 1p/19q-codeleted (WHO grade II/III); 42 astrocytic gliomas, *IDH*-wildtype, including 6 pilocytic astrocytomas (WHO grade I), 28 glioblastomas and 1 gliosarcoma (WHO grade IV); 2 diffuse midline gliomas, *H3K27M*-mutant (WHO grade IV); 8 medulloblastomas (WHO grade IV) and 19 meningiomas (WHO grade I/II/III) were analyzed.

### DNA extraction, library preparation and sequencing

We designed a DNA panel that is suited for detection of DNA mutations, InDels, LOH and CNV (target region size: 459 kbp, total target bases analyzable: 254 kbp; 57 genes, 4082 SNPs, 98.83% coverage). Genes and chromosomal regions included in the panel are listed in Suppl. Table 1. Most genes were covered using the complete coding sequence (CDS), for some genes with well-known mutations only hotspot regions were covered. Chromosomal regions were covered with commonly heterozygous SNPs. 19 genes relevant for CNV analysis were additionally covered with commonly heterozygous SNPs in intronic regions. Genes and chromosomal regions were selected in a way to be 1) typical for a specific tumor entity (diagnostic), 2) indicate response to targeted therapies in other solid cancers (targetable) and/or 3) indicate drug resistance (resistance) [14, 15]. Additionally genes involved in DNA damage response (DDR) that point to the presence of a potential hypermutator phenotype were included.

Considerations on panel size, cost-efficiency, customizability, removal of PCR duplicates and the type of starting material (FFPE) led to the use of an amplicon capture-based target enrichment system (HaloPlex HS, Agilent) and the MiniSeq instrument (Illumina). The workflow is shown in Suppl. Figure 1. Total genomic DNA was isolated from FFPE tissue slides, using the GeneRead DNA FFPE Kit (Qiagen). Microdissection was administered prior to DNA extraction to increase tumor cell content where necessary. DNA was quality-assessed with an automated electrophoresis tool (Tapestation 4200, Agilent) and quantified using Quant-iT PicoGreen dsDNA Kit (Thermo Fisher Scientific) with the minimum requirements of 50 ng to 200 ng total mass for library preparation. Library preparation was performed with an amplicon capture-based target enrichment system according to manufacturer’s guidelines (HaloPlexHS, Agilent). In short, DNA was digested with defined restriction enzymes and denatured. Target specific probe libraries including sequencing binding motives, indices, PCR oligonucleotides and molecular barcodes (for removal of PCR duplicates) were hybridized overnight to DNA targets. Uniquely barcoded targets were ligated and captured via streptavidin biotin binding. Enriched targets were amplified via PCR. Library quality and quantity was assessed using a TapeStation measurement (Agilent). Libraries were pooled and sequenced on a MiniSeq instrument (Illumina) with a theoretical average coverage of 750-fold. Flow cells were selected according to sample number, required target coverage and required read length (300 bp paired end). The sequencing run was performed in the standalone modus resulting in raw bcl files.

### Data analysis

NGS data analysis was performed with freely available, customizable tools on a Linux-based workstation. Bcl files were demultipexed (samples were separated according to indices) and converted into the fastq format with the bcl2fastq tool from Illumina, Version 1.8.4 [16]. Adaptor trimming was performed with the cutadapt tool [17] and quality checks in the standard fastq.gz format were performed with fastQC [18]. Resulting fastq files were aligned to the reference genome (GRch37) using the BWA mem tool [19] and further processed (sorted and indexed) with SAMtools 1.2 [20]. From the generated BAM-files, PCR duplicates were removed with the LocatIT tool [21]. To quantify NGS quality, percentages of target regions that were covered with a defined number of reads (1, 5, 10, 20, 30, 50, 100, 500 and 1000 reads), the deduplication rate and read number in the deduplicated BAM file were considered. Data were then ready for further processing.

For SNP/InDel calling and CNV analysis the VarScan tool (v2.4.3) [22] and SAMtools mpileup (Version 1.2) were used [20]. The VarScan 2 algorithm reads SAMtools mpileup output from tumor and normal (in our case matched blood) samples simultaneously, performing pairwise comparisons of base calls and normalized sequence depth at each position. For variant detection, VarScan somatic determines the genotype for normal and tumor samples independently based on adjustable minimum thresholds for coverage, base quality, variant frequency and statistical significance. To refine SNP calling, the VarScan fpfilter was used as a false-positive filter that removes likely false positives due to sequencing−/alignment-related artefacts [22]. Filter criteria were set by default, except for minimal variant frequency that was changed from 5 to 10%, defining the sensitivity of our SNP calling analysis. To prevent a potential issue for variant calling due to lower coverage of important diagnostic or predictive SNPs (for example *TERTp*, *IDH1/2*, *BRAF*, etc.), an additional SNP analysis with SAMtools mpileup was performed.

Vcf files were annotated by annovar [23] with additional information from databases like dbSNP, 1000Genomes Project and COSMIC as well as SIFT and PolyPhen scores to deviate possible biological relevance. Every called InDel was visually checked for plausibility using the Integrative Genomics Viewer (IGV) software [24]. LOH analysis was performed with a self-made filtering using the SNP VCF-file from VarScan somatic. To filter heterozygous SNPs, variant frequencies from normal samples between 30 and 60% were applied. Further filtering criteria were more than 20 alternative reads in the normal sample and more than 20 reads in sum in the tumor sample. A ratio of tumor variant frequency and normal variant frequency higher than 1.35 and lower the 0.67 was considered as LOH [25].

**Fig. 1:**
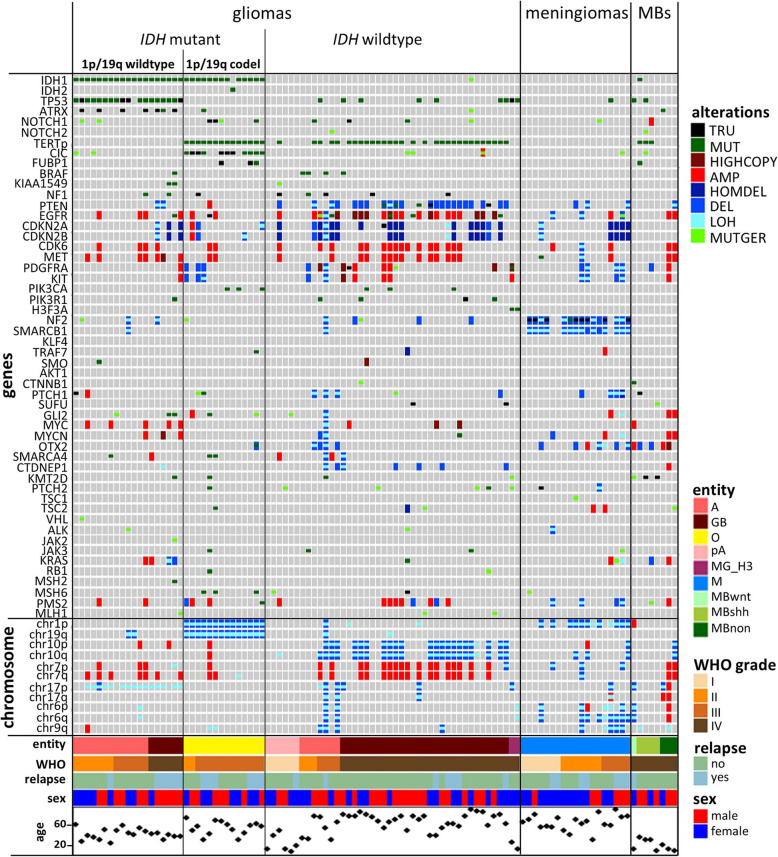
Overview of the DNA Panel NGS results. Molecular alterations per case are illustrated in form of an oncoprint figure [27]. The figure contains information on the histological diagnoses according to WHO 2016 criteria, molecular alterations, the presence of relapse as well as age and sex of the patients. A: astrocytoma, GB: glioblastoma, O: oligodendroglioma, pA: piloytic astrocytoma, MG_H3: diffuse midline glioma, *H3K27M*-mutant; M: meningioma; MB: medulloblastoma, wnt: WNT-activated, shh: SHH-activated, non: non-WNT/SHH. TRU: truncating variation probably leading to a loss of function, MUT: somatic missense variation, HIGHCOPY: highcopy amplification, AMP: amplification, HOMDEL: homozygous loss, DEL: deletion, LOH: loss of heterozygosity, MUTGER: germline SNP with minor allele frequencies for Europeans (non-Finnish) < 0.01 and number of homozygotes SNPs < 5

**Fig. 2:**
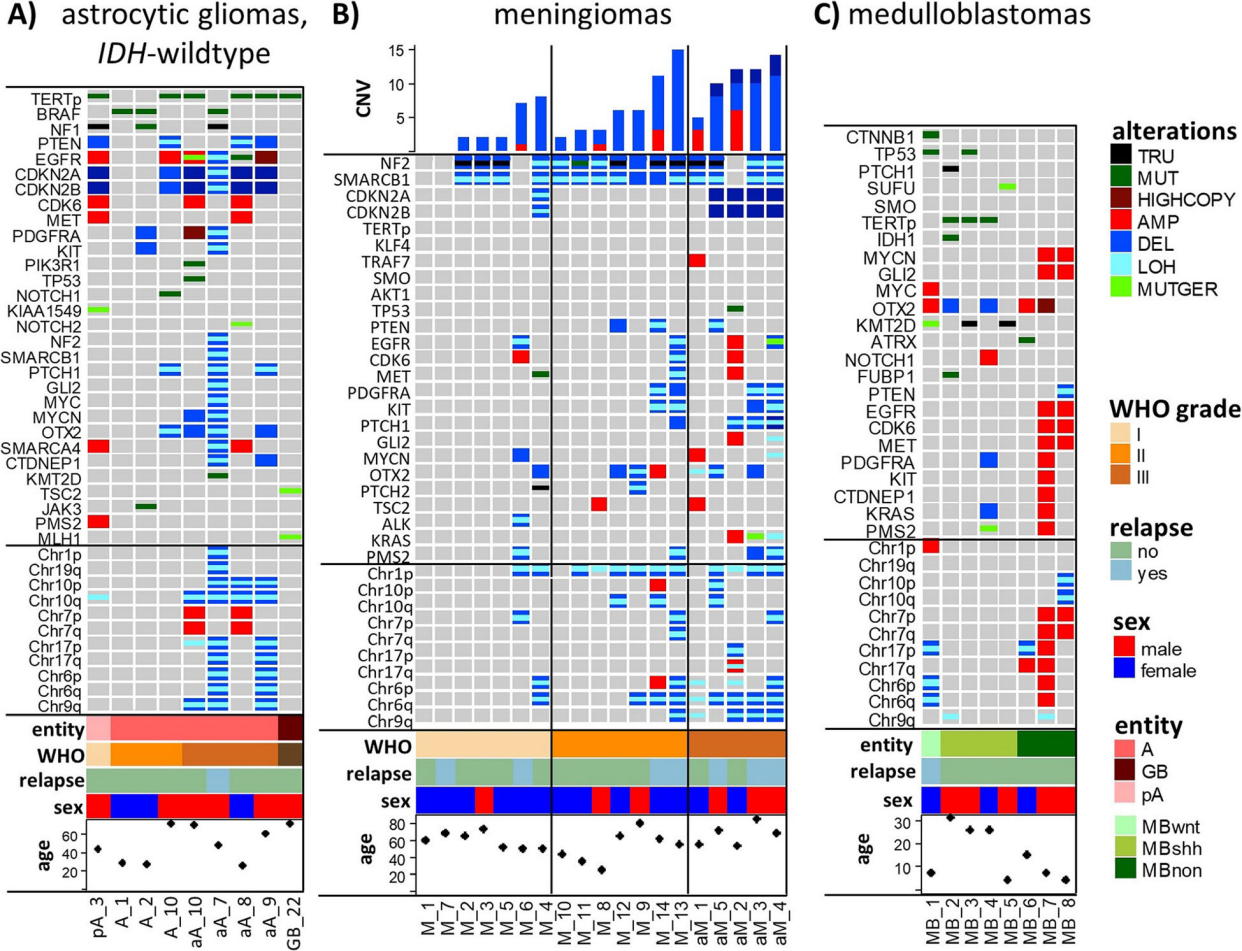
Illustration of DNA panel results conveying diagnostic and clinically relevant information in selected cases of gliomas (**a**), meningiomas (**b**) and medulloblastomas (**c**). For abbreviations, compare legend to Fig. 1

To identify somatic CNV, VarScan copynumer and copyCaller with matched blood or FFPE normal samples were used and data were visualized with the R bioconductor package DNAcopy [26]. CNV results for broad chromosomal regions and genes were generated separately. Baseline was assessed individually for every sample, based on sorting segments according to their size. LOH analysis results were also considered. For CNV analysis of chromosomal regions baseline plus/minus 0.4 was considered as balanced. Chromosomal regions with gene dosage increases of more than + 0.4 were declared as amplified, whereas regions with gene dosage decreases of more than − 0.4 were considered as lost. CNV alterations of genes were considered as relevant starting with at a cut-off of 0.5. Gene segments with dosages more than 1 above the baseline were considered as highcopy amplifications and those with dosages below 1 as homozygous losses. All results generated in this way were visualized with the oncoprint tool provided by the complex heatmap package from R [27] (Figs. 1 and 2).

### Validation methods

Classical diagnostic markers (*IDH1/2*, *H3F3A*, *BRAF* and 1p/19q LOH) were assessed according to quality-controlled protocols established in our lab [28]. Briefly, hotspot mutations in *IDH1/2*, *H3F3A* and *BRAF* were analyzed with direct sanger sequencing after PCR-based amplification of the locus with PCR. 1p/19q LOH was assessed by microsatellite PCR. To validate other SNPs and small InDels detected with the NGS approach, we used direct sanger sequencing following region-specific PCR. Oligonucleotides are listed in Suppl. Table 2. *TERTp* mutations were detected using the oligonucleotides and protocol described in [29]. PCR reactions were performed using the HotStar Taq DNA Polymerase (Qiagen) and sequencing with the BigDye Terminator v1.1 Cycle Sequencing Kit (Thermo Fisher Scientific) on the SeqStudio Genetic Analyzer (Thermo Fisher Scientific), all according to manufacturers’ protocols. For validation of *EGFR* highcopy amplification and *CDKN2A* homozygous deletion, a target specific quantitative PCR was performed as described in [30]. For further validation of our CNV analysis, an OncoScan CNV Assay (Thermo Fisher Scientific) was performed externally as a contract work for 8 selected cases (IMGM Laboratories, Munich). 7 cases were selected because of their high amount of CNV/LOH variations and 1 case lacking CNV/LOH variations was included as a negative control. To determine assay performance, test accuracy, sensitivity (true positive rate) and specificity (true negative rate) were calculated according to [31].

## Results

### Precision and reliability of the targeted NGS approach

We analyzed 104 CNS tumors classified according the WHO 2016 classification criteria [1]. Entities included and commonly occurring variations for each entity are summarized in Table 1. Variations could be detected in the range of the expected frequencies [1]. Sequencing results of all cases together with additional clinical information are listed in Suppl. Table 3 and visualized in an oncoprint figure (Fig. 1). DNA panel sequencing was robust with a mean of 87.6% target regions covered with at least 10 reads for all cases analyzed (Suppl. Table 3). Coverages showed a weak positive correlation to the DNA quality value (DIN) measured with an automated electrophoresis tool (TapeStation, Agilent). The few cases (8) with coverages below 80% also had low DIN values (< 3.5) (Suppl. Figure 4).

**Table 1:** Overview of all cases analyzed and entity specific alterations with frequencies

Entity	#	Aberrations	#	Frequency
Astrocytic glioma, *IDH*-mutant (WHO grade II/III/IV)	19	IDH1/2	19	100%
		TP53	18	95%
		ATRX	7	37%
		CDKN2A HomDel	2	11%
Oligodendroglioma, *IDH*-mutant 1p/19q-codeleted (WHO grade II/III)	14	IDH1/2	14	100%
		1p/19q LOH	14	100%
		TERTp	14	100%
		CIC	11	79%
		FUBP1	4	29%
Astrocytic glioma, *IDH*-wildtype (WHO grade I/II/III/IV)	42	TERTp	30	71%
		10 DEL / 7 AMP	14	33%
		EGFR Highcopy	9	21%
		TP53	9	21%
		NF1	5	12%
		BRAF	4	10%
Diffuse midline glioma, *H3K27M*-mutant (WHO grade IV)	2	H3F3A	2	100%
		TP53	2	100%
Meningioma (WHO grade I/II/III)	19	NF2	15	79%
		SMARCB1	15	79%
		1p DEL	12	63%
		CDKN2A HomDel	4	21%
		TERTp	0	0%
Medulloblastoma (WHO grade IV)	8	TERTp	3	38%
		TP53	2	25%
		OTX2 Highcopy	1	13%
		CTNNB1	1	13%
		Monosomy 6	1	13%
		Isochromosome 17	1	13%

AMP: amplification, DEL: deletion, Highcopy: highcopy amplification, HomDel: homozygous loss, LOH: loss of heterozygosity

As prior to panel sequencing for all 104 cases molecular information had been obtained for targets such as *IDH1*, *IDH2*, *BRAF*, *H3F3A* mutation and 1p/19q loss by means of single molecular assays within the routine diagnostic procedure, this information could be directly compared. All other commonly occurring variations within the different entities were subsequently validated by use of direct sanger sequencing and quantitative PCR. For quality assurance approval of our assay according to the ILAC (DAkkS) standards for inspection bodies (ISO/IEC 17020) [32, 33], we defined a QC (quality control) cohort of 17 cases comprising 4 astrocytic gliomas, *IDH*-mutant (WHO grade II/III/IV); 2 oligodendrogliomas, *IDH*-mutant and 1p/19q-codeleted (WHO grade III); 5 astrocytic gliomas, *IDH*-wildtype, including 3 glioblastomas (WHO grade IV) and 1 diffuse midline glioma, *H3K27M*-mutant (WHO grade IV); 4 meningiomas (WHO grade I/II/III) and 2 medulloblastomas (WHO grade IV), 1 WNT-activated and 1 SHH-activated. For these 17 cases we validated the majority of all detected SNPs and small InDels. To validate our CNV and LOH results, DNA panel results of 8 out of the 17 cases were compared to an OncoScan CNV analysis. Validations results for all cases analyzed are summarized in Suppl. Table 4. Examples of the validation procedures are shown for an oligodendroglioma, *IDH*-mutant and 1p/19q-codeleted in Suppl. Figure 2 and for a glioblastoma, *IDH*-wildtype in Suppl. Figure 3.

Overall performance of our NGS panel was determined by sensitivity and specificity (Table 2). For the classical diagnostic biomarkers *IDH1/2* mutation, 1p/19q loss, *BRAF* and *H3F3A* mutation, sensitivity and specificity were both 100%. For *TERTp* mutation, *EGFR* highcopy amplification and *CDKN2A* homozygous deletion (recommended as upcoming diagnostic markers in the cIMPACT now update 3 [2] and in a novel grading system for *IDH*-mutant astrocytomas [3]) clinical sensitivity and specificity was equally 100%. Thus, these main diagnostic markers can be analyzed with the identical sensitivity and specificity in our NGS approach as compared to single gene assay techniques.

**Table 2:** Validations performed for quality control according to the ILAC (DAkkS) standards for inspection bodies (ISO/IEC 17020)

	Abberation	# Cases	# Validations	Validation method	Sensitivity	Specificity
**Single assay validation**	IDH1/2	34	68	Sanger seq.	100%	100%
	1p/19q LOH	15	35	Microsatellite PCR	100%	100%
	BRAF V600	2	3	Sanger seq.	100%	100%
	H3F3A	2	3	Sanger seq.	100%	100%
	TERTp	47	50	Sanger seq.	100%	100%
	CDKN2A HomDel	8	11	quantitative PCR	100%	100%
	EGFR Highcopy	12	14	quantitative PCR	100%	100%
**QC cohort**	other SNPs / InDels	17	28	Sanger seq.	100%	n.d.
	other LOH	8	28	Oncoscan Array	90%	97%
	all CNVs	8	47	Oncoscan Array	94%	97%
**Overall performance**					**97%**	**98%**

DNA panel results were compared to quality-controlled single assays in our routine diagnostic lab for the established molecular biomarkers *IDH1/2*, *BRAF* and *H3F3A* mutation as well as 1p/19q codeletion. *TERTp* mutations, *CDKN2A* homozygous deletions and *EGFR* highcopy amplifications were validated by direct sanger sequencing or quantitative PCR. Intensified quality assurance approval was performed in a cohort of 17 tumors. In these cases, the majority of detected SNPs and small InDels were reanalyzed using direct Sanger sequencing. Other LOH results and CNV results were reanalyzed using an OncoScan CNV analysis. Comparison of DNA panel and validation results yielded excellent sensitivity and specificity. n.d.: negative results were not validated, QC: quality control cohort

Other SNPs and small InDels validated in our QC cohort achieved a sensitivity of 100% (in this context we abstained from validating negative results due to the multitude of alterations). For our LOH analyzes a sensitivity of 90% and specificity of 97%, and for CNV analysis a sensitivity of 94% and specificity of 97% were achieved. To determine diagnostic test accuracy, we performed NGS panel analysis in two independent cycles with 3 cases out of the 17 obtaining identical results. Here, we achieved 100% accuracy for the SNP and small InDels calling analysis, for LOH analysis as well as for the CNV analysis.

Taken together, our targeted NGS approach proved precise and reliable with 100% accuracy, 97% sensitivity and 98% specificity.

### Diagnostic implications in gliomas

77 astrocytic and oligodendroglial tumors were analyzed on our new platform (for detailed information see above). While the initial 42 cases had been evaluated for validation purposes, the subsequent 35 cases were analyzed within a diagnostic and clinical context. Out of these 35 cases, 9 received a refined diagnosis or even reclassification after consideration of the molecular data resulting from NGS panel sequencing. Thus, 26% of patients (more than every fourth) benefited from in-depth molecular analysis. Context situations of diagnosis refinement were as follows:

Our cohort contained 3 diffuse and 4 anaplastic *IDH*-wildtype astrocytomas that are considered as a provisional entity for which diagnosis is discouraged. Indeed, panel sequencing enabled reclassification of these cases. 4 tumors (A_10, aA_8, aA_9 and aA_10; Fig. 2a) contained prognostically relevant alterations as highlighted in the recent cIMPACT-NOW update 3 [2]. By the presence of *TERTp* mutations, *EGFR* highcopy amplification and/or combined gains or losses of whole chromosome 7 and 10 these tumors could be reclassified as diffuse astrocytic gliomas, *IDH*-wildtype, with molecular features of glioblastoma. Thereby, the tumors could be assigned to a WHO grade IV, impacting subsequent therapeutic strategies. The other 3 *IDH*-wildtype astrocytomas (A_1, A_2, aA_7; Fig. 2a and Suppl. Figure 5) were reclassified due to miscellaneous reasons:

Tumor A_1 showed no additional alterations except for a *BRAF* (p.V600E) mutation. Close histological reevaluation revealed irregularly arranged neuronal elements at the periphery of the lesion, highlighted by antibodies to synaptophysin and neurofilament. Moreover, CD34-immunostaining identified peritumoral satellite cells. These findings led to the revised integrated diagnosis of ganglioglioma (WHO grade I).

Tumor A_2 was the case of a 27 years old patient with an intra- and periventricular brain lesion involving the basal ganglia. Detection of a *BRAF* V600E mutation next to mutations in *NF1* and *JAK3* as well as deletions of *KIT* and *PDGFRA* led to the diagnosis of a pediatric-type low-grade glioma or ganglioglioma. A parallel 850 k methylation array analysis also achieved the highest score (0.51) for low-grade gliomas, in particular the subclass of gangliogliomas.

Tumor aA_7 exhibited multiple losses of chromosomal regions, homozygous deletion of *CDKN2A/B* and a particular mutation of *BRAF* (p.D594G) by NGS analysis. With these findings -even in absence of typical histological features as lipidized cells, a pericellular reticulin network and co-expression of neuronal markers- we reclassified the tumor as anaplastic pleomorphic xanthoastrocytoma (WHO grade III). Though, in pleomorphic xanthoastrocytomas most of the *BRAF* mutations are of the V600E type, exceptions as in the present case are well documented [34]. DNA methylation-based analysis of aA_7 showed elevated scores for pilocytic astrocytoma and BRAF-mutant low-grade glioma but could not finally classify the lesion.

Apart from reclassifying *IDH*-wildtype astrocytomas, panel sequencing contributed to clarifying the difficult differential diagnosis between reactive gliosis and glioma infiltration in biopsy samples of limited glial cell density and confined p53 immunoreactivity. In GB_22 (Fig. 2a), detection of a *TERTp* mutation pointed to a neoplastic origin of the increased glial cell content resulting in the diagnosis of the infiltration zone of an *IDH*-wildtype glioblastoma.

Finally, panel sequencing identified cases with unusual molecular features and clinical course. On magnetic resonance imaging (MRI) scan, a frontal lobe tumor presented as a poorly defined mass with T2-FLAIR hyperintensity and only very minor gadolinium enhancement, so that a low-grade glioma was suspected. Microscopic analysis disclosed an *IDH*-wildtype astrocytic glioma with only moderate cell density and low Ki67-index (3%), first classified as pilocytic astrocytoma (pA_3, Fig. 2a). However, in the small specimens, a decisive tumor classification appeared difficult since the morphological features were compatible with pilocytic astrocytoma, but also other pediatric-type gliomas or even low cellular areas of glioblastoma. The absence of *KIAA1549-BRAF* fusion did not necessarily exclude the diagnosis of a pilocytic astrocytoma. Panel sequencing enabled the detection of a *TERTp* mutation, a loss of function mutation in the *NF1* gene, a homozygous deletion of *CDKN2A*, and LOH on chromosome 10q along with deletion of *PTEN.* This would be a rather unusual molecular constellation for pilocytic astrocytoma. Instead, combined mutations in *TERTp*, *PTEN* and *NF1* are described for high-grade gliomas and are associated with an unfavorable prognosis [35, 36]. Not surprisingly, this patient suffered from tumor progression at already 9 months after the first surgery, despite gross total resection.

### Prognostic refinement in meningiomas

We analyzed 19 meningiomas, graded by conventional histology as 7x WHO grade I, 7x WHO grade II and 5x WHO grade III (Fig. 2b and Suppl. Table 3). The main and most consistent variations detected, as expected, were *NF2* truncating mutations and/or deletions/LOH of the *NF2* gene locus [37]. As can be seen from Fig. 2b, complexity of detected CNVs was heterogeneous within the grades and a high CNV content was not exclusively reserved to WHO grade III meningiomas, but could be also observed in individual WHO grade I and II lesions. In the WHO grade I tumor group, for example, 2 meningothelial meningiomas (M_4 and M_6, Fig. 2b) showed 8 and 7 CNV variations, respectively, compared to 0 to 2 variations detected in the other WHO grade I tumors. One of these cases (M_4, WHO grade I) had a 1p and an additional *CDKN2A/B* loss, both described as prognostic unfavorable molecular alterations [1, 6]. Indeed, the respective patient exhibited a poor clinical course with tumor progression after 17 months, despite gross total tumor resection (Simpson grade 1) and a tumor size of only 41 mm without sinus infiltration.

In the WHO grade II group, we identified a tumor with a very high number of 14 CNVs (M_13, Fig. 2) and a prognostically unfavorable 1p loss. Also for that case, the respective clinical course was poor with a tumor progression after 10 months, despite a gross total tumor resection (Simpson grade 1) and a tumor size of 88 mm without sinus infiltration.

Thus, the detection of prognostically unfavorable alterations and the appreciation of the complexity of CNVs can help to identify patients with a higher risk of recurrence in the group of benign WHO grade I and prognostically heterogeneous WHO grade II meningiomas.

### Subtyping of medulloblastomas

We analyzed 8 medulloblastoma that had been subtyped with an immunohistochemical approach [10]: 1 WNT-activated, 4 SHH-activated (3 *TP53*-wildtype and 1 *TP53*-mutant) and 3 non-WNT/non-SHH-activated cases (Fig. 2c and Suppl. Table 3). With our targeted DNA panel sequencing approach, subgroups could be retraced in all cases by identifying alterations of typical pathway genes:

The WNT-activated medulloblastoma (MB_1) showed characteristic variations specific for this subgroup, like a *CTNNB1* mutation and chromosome 6 monosomy [8]. In the SHH-activated medulloblastoma cases, 2 SHH pathway specific mutations in *SUFU* (MB_5) or *PTCH1* (MB_2) were detected. 3 of the 4 SHH-activated medulloblastomas harbored a *TERTp* mutation (MB_2, MB_3, MB_4), which has also been described as a variation specific for SHH-activated medulloblastomas [1, 38]. Interestingly, one of the cases (MB_2) was a rare SHH-activated medulloblastoma showing an *IDH1*:p.R132C mutation. Of the 4 cases described harboring such a mutation 3 fell into the SHH-activated subgroup of medulloblastomas [12]. Non-WNT/SHH-activated medulloblastomas are less well characterized in terms of pathway activation [39]. Nevertheless, there are some characteristic CNVs for this subgroup detected with our DNA panel analysis, like isodicentric chromosome 17q (MB_6), chromosome 17q amplification (MB_7) and chromosome 7 amplification (MB_7 and MB_8) [12].

### Identification of actionable mutations for individualized treatment approaches

Genes were referred to as targetable or resistance-mediating according to ICGC (the international cancer genome consortium) or CIVIC (clinical interpretations of cancer variants from the McDonnell Genome Institute at Washington University School of Medicine) if there was proven/consensus association in human medicine or evidence from clinical trials, case reports or other primary data in brain tumors or other cancer entities [14, 15] (Suppl. Table 1).

**Fig. 3:**
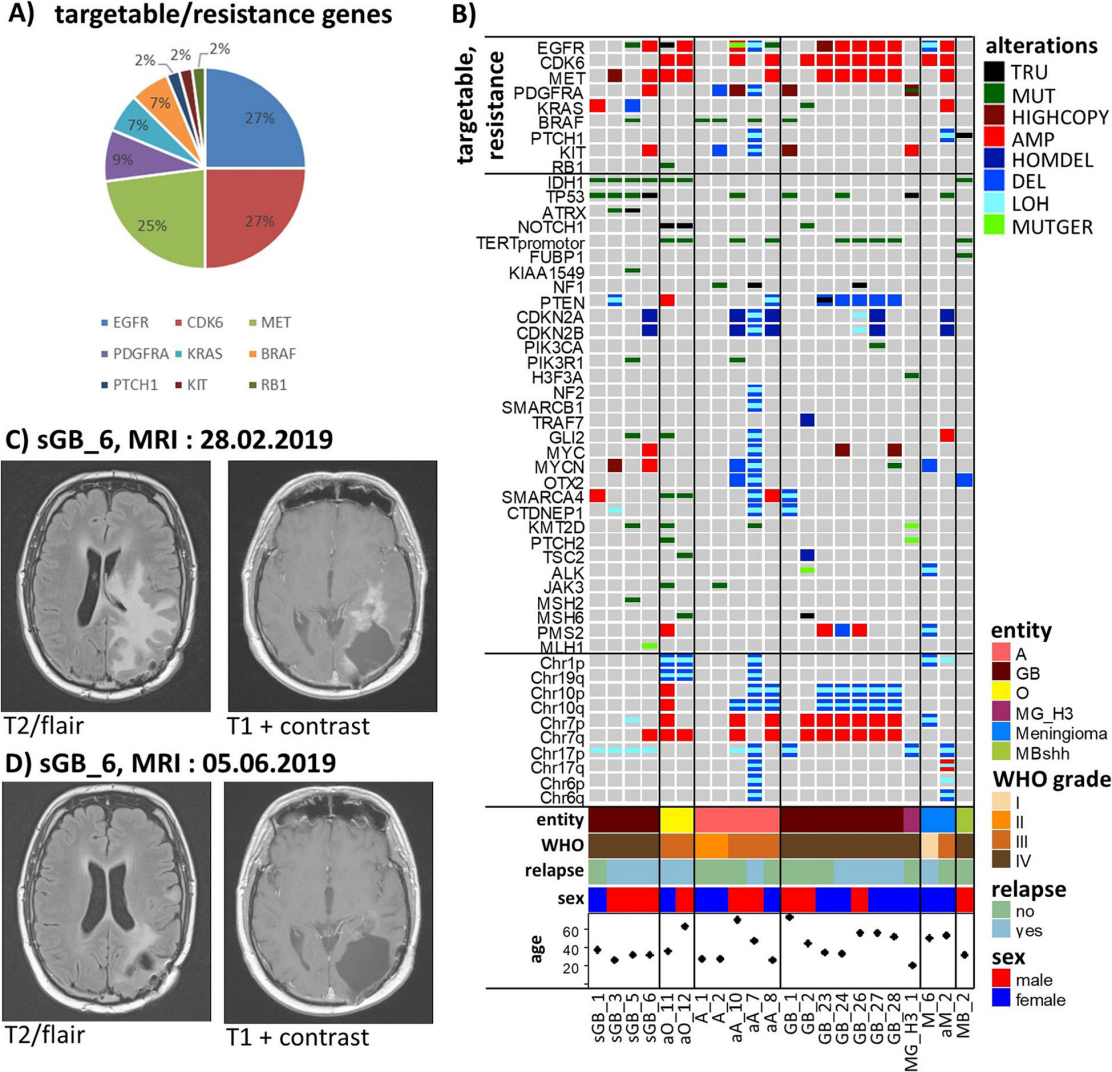
Targeted therapy based on DNA panel sequencing results. (**a**) Frequencies of putatively targetable and therapy resistance-mediating variations within the clinically requested cohort. (**b**) Shown is the full spectrum of molecular alterations in all cases containing targetable and resistance-mediating variations in form of an oncoprint fig [27]. For abbreviations, compare legend to Fig. 1. (**c**) MRI of sGB_6 shows one region of extended hyperintensity (FLAIR) and a further region with notable contrast enhancement in T1 before starting individualized therapy. (**d**) After two cycles of therapy, hyperintensity (FLAIR) was clearly diminished and contrast enhancement almost completely disappeared, indicating partial response. Images were chosen on the levels of maximum lesions, as it would also be done for evaluation of the neuroradiological RANO criteria, that are standard for the evaluation of tumor treatment responses in gliomas [40]

Of the 104 cases that were analyzed on our platform 60 cases were used for establishing the assay and for validation purposes. 44 cases were analyzed on clinical request. Of note, 22 out of these 44 cases (50%) showed putatively targetable or resistance-mediating variations with *EGFR*, *CDK6* and *MET* aberrations most frequently detected (Fig. 3b).

After careful revision by a group of clinicians (neurooncologist, neurosurgeon, medical oncologist) 3 patients were chosen for an individualized therapy based on molecular data generated by our NGS approach (Table 3). Only patients in a relatively good condition for which standard therapeutic approaches were exhausted were selected from the clinical side. These patients’ tumors then had to harbor molecular variations with clear therapeutical relevance and sufficient variant frequencies from the neuropathological side explaining the low number of patients included into this algorithm so far. The case histories of the three patients were as follows:

**Table 3:** Information on patients that received a targeted therapy based on DNA panel sequencing results

Patient ID	Entity	Therapeutic	Description	Target	Response
sGB_6	Secondary glioblastoma, *IDH*-mutant (WHO grade IV)	Cabozantinib	Tyrosine kinase inhibitor	MET AMP, KIT AMP	yes
MB_2	Medulloblastoma, SHH-activated/*TP53*-WT (WHO grade IV)	Vismodegib/ sonidegib	Sonic hedgehog pathway inhibitor	PTCH1 stopgain with a frequency of 92%	yes
aA_8	Anaplastic astrocytoma, *IDH*-wildtype (WHO grade III)	Abemaciclib	CDK4/6 inhibitor	CDK6 AMP	no
	*reclassified as:*				
	Diffuse astrocytic glioma, *IDH*-wildtype, with molecular features of glioblastoma (WHO grade IV)				

Patient 1 was a 33-year old male (sGB_6) diagnosed with astrocytoma, *IDH*-mutant (WHO grade II) and relapse with a secondary glioblastoma without *MGMT* methylation. Based on panel sequencing results with amplification of *MET* and *KIT* and tumor board decision, cabozantinib was initiated in a dose of 100 mg daily in the third relapse of the disease. The patient showed a very good clinical response with a recovery to almost normal and a good partial response on MRI which was sustained for 6 months (Fig. 3, c-d). Response was achieved without further surgery and solely based on the targeted therapy. After 6 months of Cabozantinib, the patient progressed clinically and on MRI. Treatment was terminated and the patient died another 3 months later in hospice care. In summary, overall survival with glioblastoma was 20 months, and progression free survival with Cabozantinib was 6 months.

Patient 2 was a 31-year old male (MB_2) diagnosed with medulloblastoma, SHH-activated and *TP53*-wildtype (WHO grade IV), and presenting with an unusual relapse involving the nuchal lymph nodes but not the primary site. Lymph nodes were biopsied and histology proved dissemination of a medulloblastoma. Based on panel sequencing results with a *PTCH1* loss of function mutation combined with a LOH (variant frequency: 92%) and tumor board decision, vismodegib was initiated in a dose of 150 mg daily in combination with temozolomide. The patient showed a good partial response and terminated temozolomide after 6 months and vismodegib after 12 months of treatment. A year later he presented with enlarged lymph nodes in his right axilla and mediastinum and with a local relapse at the primary site in the cerebellum. The axillar and mediastinal lymph nodes were irradiated with 54 Gy, and temozolomide was combined with sonidegib 200 mg daily, based on the superior pharmacokinetic data for sonidegib in comparison to vismodegib. The patient again showed a good partial response in his lymph nodes and a complete response at his primary site in the cerebellum after 3 months of treatment. In summary, overall survival was 43 months after diagnosis, progression free survival in the first relapse under treatment with vismodegib and temozolomide was 15 months, and a second partial response was noted under sonidegib plus temozolomide, which was sustained at the time of this report (10 months).

Patient 3 was a 26-year old female patient (aA_8) diagnosed with anaplastic astrocytoma, *IDH* wildtype (WHO grade III) without *MGMT* promoter methylation. By DNA panel sequencing the tumor was reclassified as diffuse astrocytic glioma, *IDH*-wildtype, with molecular features of glioblastoma (WHO grade IV, see above). Based on panel sequencing with a *CDK6* amplification and decision of our interdisciplinary tumor board, abemaciclib in combination with temozolomide was initiated in the fourth relapse. At the beginning of abemaciclib, the patient was in a good self-containing state. She deteriorated slowly during abemaciclib and temozolomide in her MRI and clinical status, and the therapy was terminated 3 months later. In summary, overall survival was 33 months, and abemaciclib and temozolomide did not generate an objective response or stabilization of disease. Possible reasons for the lack of response in this case may be the late administration of the targeted agent in the fourth relapse and a very large tumor volume on MRI.

Taken together, at least the first two case reports indicate that selected patients may benefit from molecularly driven approaches as derived from our panel sequencing assay even when standard therapies are exhausted.

## Discussion

The rapid evolution of meaningful molecular markers in brain tumor classification and therapy poses challenges to many neuropathology labs. When thinking about a suitable way to address the increasing number and complexity of alterations that have to be tested for, we made the following considerations: We wanted to establish a single platform approach that would allow for the parallel detection of diagnostically and therapeutically relevant markers. The approach should be cost-efficient, on an in-house platform and quality-controlled. It should fit into our lab’s molecular workflow and present with acceptable turnaround times to not delay patients’ clinical management. The NGS panel sequencing assay presented here fulfills these requirements. Our customized amplicon capture-based based approach allows detection of molecular alterations with high specificity and accuracy. Accuracy was 100%, sensitivity 97% and specificity 98%, leading to quality assurance approvement according to the ILAC (DAkkS) standards for inspection bodies (ISO/IEC 17020) [32, 33]. It can be run on the relatively cost-efficient Illumina MiniSeq platform with a 5-day workflow from DNA isolation to bioinformatic analysis and the molecular report (Suppl. Figure 1).

In terms of the diagnostic surplus value we could show that NGS panel sequencing leads to diagnostic refinement or reclassification in about a fourth of gliomas, identifies molecularly suspect meningiomas with an unfavorable clinical course and allows for the precise subclassification of medulloblastomas. Moreover, it identifies actionable mutations of clinical use in a relevant fraction (50%) of patients that can be exploited for successful targeted approaches.

A number of NGS-based approaches for brain tumor diagnostics have been reported so far, showing similarities but also differences to our findings [13, 41–46]. Most of the publications emphasize the diagnostic benefit as well as the future surplus value for targeted therapies. Interestingly, one of the other panel sequencing approaches evaluating data on a large series of 433 gliomas [44] -identically to our observations- reports on a fraction of about a fourth of cases with refined diagnoses. In terms of the composition of genetic alterations included and the technical realization, however, there are large differences between the reported assays. The group in [13], for example, uses a similar library preparation technique as we do, but runs a larger panel on a NextSeq 500 platform that is primarily focused on the detection of actionable mutations. The group in [45] employs a completely different library preparation method and sequencing technique based on the Ion Torrent (PGM) platform.

The assay presented here is unique as it also allows for the parallel assessment of SNPs, InDels, CNV and LOH with the latter based on the comparison of often-mutated SNPs between tumors and matched normal controls. This widens the functionality of our panel in more complex diagnostic context situations such as medulloblastoma subclassification or identification of cIMPACT-NOW update 3 alterations. The necessity to analyze matched patients’ blood samples may be beneficial also in another respect. It allows for the sure identification of somatic variations without any possible germline contaminations what we would consider recommendable for this type of analysis anyway, independent on whether LOH analyzes are performed or not. In our cohort germline variations misinterpreted as somatic mutations would have led to false positive results in 37% of the cases. Incidentally observed, tumor-relevant germline variations can be reported in collaboration with human genetics according to mandatory legal requirements. Another significant difference of our assay in comparison to others is the cross-entity suitability including alterations relevant to gliomas, meningiomas and medulloblastomas. In our perception this has a number of advantages, particularly in smaller labs with fewer NGS requests. First, in terms of efficiency, it allows us to focus on only one library preparation approach and to smoothly pool the libraries on the same flow cell. This decreases turnaround times as every NGS order fills the flow cell independently of the underlying diagnosis. Moreover, we have also included alterations like *C19MC* amplification in our panel. Having this in store as a single molecular, e.g. FISH assay, would not be cost-efficient as due to the very low commissioning frequency of the assay, probes would expire before they are used up. By inclusion in our comprehensive panel approach we can hold the marker in store and run the analysis whenever indicated. Finally, the combination of multiple alterations in our assay supports differential diagnoses and has synergistic effects in terms of alterations that contain both diagnostic and predictive informational content.

However, there are also limitations to our approach. *MGMT* promoter methylation is a marker that cannot be included in our setting and has to be tested separately. Also, we cannot detect gene fusions, like e.g. *KIAA1549:BRAF, RELA:C11ORF95, YAP1, FGFR, MYB1* in a diagnostic or *NTRK* in a predictive context [47–52]. We are currently working on an RNA-based NGS approach that similarly to the here reported DNA panel will cover a larger spectrum of entities with a focus on pediatric brain tumors. As outlined above, we performed the whole NGS data analysis with freely available, customizable tools on a Linux-based workstation. Set-up of theses algorithms was a time-consuming investment and may be a hurdle for those labs thinking about starting with similar workflows. We also tried a couple of tools that were available by commercial suppliers. These may be well-suited in certain context situations. However, we felt that for a full support of our analysis and for flexible adaptation to our evaluation needs the use of custom-made scripts was indispensable. Once up and running these scripts multiply the informational content and help to exploit the whole potential of the method.

In conclusion, we here describe a targeted NGS-based DNA panel approach that comprehensively addresses the most relevant alterations in molecular neurooncology and may be suited also for implementation in smaller neuropathology labs. Our assay has been extensively validated and proven to convey diagnostic and clinical surplus value. As additional markers can be easily included, the assay can be adapted to the specific requirements of local brain tumor centers and appears future-proof in terms of upcoming revisions of the WHO classification.

## Supplementary information

**Additional file 1: Supplementary Table 1.** Genes and chromosomal regions included in the targeted DNA panel. Most genes were covered using the CDS, for some well-known mutations only hotspot regions were targeted with SNP/small InDel calling being performed (SNP). Chromosomal regions were covered with commonly heterozygous SNPs for LOH and CNV analyzes. Genes included in LOH and CNV analysis were additionally covered with commonly heterozygous SNPs in intronic regions. Variations of genes and chromosomal regions may either be characteristic for a specific tumor entity (diagnostic), indicate actionable mutations for targeted therapies (targetable) and/or drug resistance (resistance) or be associated with impaired DDR. CDS: complete coding sequence, CNV: copy number variations, DDR: DNA damage response, LOH: loss of heterozygosity, SNP: single nucleotide variant.

**Additional file 2: Supplementary Table 2.** Oligonucleotides used for validation purposes.

**Additional file 3: Supplementary Table 3.** Tabular survey of all panel sequencing results. Validation cases and clinically requested cases are indicated, also the 17 cases that were used for intensified quality control (QC cohort). HOMDEL: homozygous loss, HIGHCOPY: highcopy amplification, TRU: truncating variation probably leading to a loss of function, MUT: somatic missense variations, LOH: loss of heterozygosity, DEL: deletion, AMP: amplification, MUTGER: germline SNP with minor allele frequency European (non-Finnish) < 0.01 and number of homozygotes SNPs < 5, DELp/AMPp/LOHp: partial variation of a chromosomal arm.

**Additional file 4: Supplementary Table 4.** Tabular survey of validation results to determine DNA panel sequencing performance. SNPs and InDels were validated with direct Sanger sequencing, 1p/19q codeletions with microsatellite PCR analysis, and homozygous deletion of *CDKN2A* as well as highcopy amplification of *EGFR* with target specific quantitative PCR. Medulloblastoma subgroups were validated by comparison to immunohistochemically determined subgroups with antibodies against ß-Catenin, Yap1, p75-NGFR and OTX2 [10]. InDel: small insertion/deletion, nd: not determined, RET: retention, SNP: single nucleotide variant, WT: wildtype.

**Additional file 5: Supplementary Figure 1.** 5-day DNA panel sequencing workflow.

**Additional file 6: Supplementary Figure 2.** Validation example for an anaplastic oligodendroglioma, *IDH*-mutant and 1p/19q-codeleted (aO_1). DNA panel result of the IDH1:p.R132H mutation visualized in IGV (A) and corresponding direct sanger sequencing (B). DNA panel results of LOH analysis using commonly occurring SNPs compared to matched blood (C). Corresponding LOH analysis using microsatellite analysis with fluorescence marked oligonucleotides compared to matched blood (D). The results for one marker on chromosomal arm 1p (D1S513) and one on 19q (D19S572) are shown exemplarily. IGV: integrative genomic viewer, LOH: loss of heterozygosity.

**Additional file 7: Supplementary Figure 3.** Validation example for the CNV analysis in a case of glioblastoma, *IDH*-wildtype (GB_17). DNA panel results of the CNV analysis for chromosomal regions (A) and genes (B) were compared to a corresponding OncoScan CNV analysis (C). CNV: Copy number analysis.

**Additional file 8: Supplementary Figure 4.** Correlation of coverage with DNA quality. Percentages of target regions covered with at least 10 reads were plotted against the DIN value on a tumor by tumor basis. DIN values were measured with an automated electrophoresis tool (Tapestation 4200, Agilent). Higher DIN values indicate an intact, not degraded DNA and lower DIN values a degraded DNA of low quality. A weak (> 0.3) positive correlation between coverage and DIN value was observed indicating a dependency of coverage on DIN value.

**Additional file 9: Supplementary Figure 5.** Preoperative magnetic resonance imaging (MRI) and histology of the *IDH*-wildtype astrocytomas A_1, A_2 and aA_7. (A) A_1. MRI shows hyperintensity in FLAIR without contrast enhancement in T1 in the dorsal region of right insula, compatible with a low-grade glioma. Microscopic examination displayed a neuroepithelial lesion of moderate cell density and only low nuclear pleomorphism. Both GFAP and Synaptophysin were strongly expressed. CD34 immunoreactivity was prominent and highlighted peritumoral satellite cells. (B) A_2. MRI shows several contrast enhancing lesions on both sides of the midline with corresponding FLAIR hyperintensity. Histologically, the lesion exhibited features of a low-grade glioma in a slightly fibrillary background. Cellularity was moderate and mitoses or nuclear atypia of higher degree were absent. Singular entrapped neuronal cells were considered to be pre-existing. (C) aA-7. MRI shows a region of corresponding hyperintensity (FLAIR) and contrast enhancement (T1). Histology revealed a highly cellular astrocytic glioma with moderate pleomorphism, variable cell morphology, sometimes fusiform cells and marked aggregation of lymphocytic infiltrates.

## Data Availability

All data generated or analyzed during this study are included in this published article (and its supplementary information files). Raw data are available from the corresponding author on reasonable request.
