# Supplementary figures and images for: A comprehensive DNA panel next generation sequencing approach supporting diagnostics and therapy prediction in neurooncology
Source: Acta Neuropathol Commun. 2020 Aug 5;8:124. doi: 10.1186/s40478-020-01000-w (PMC7405456; doi:10.1186/s40478-020-01000-w)

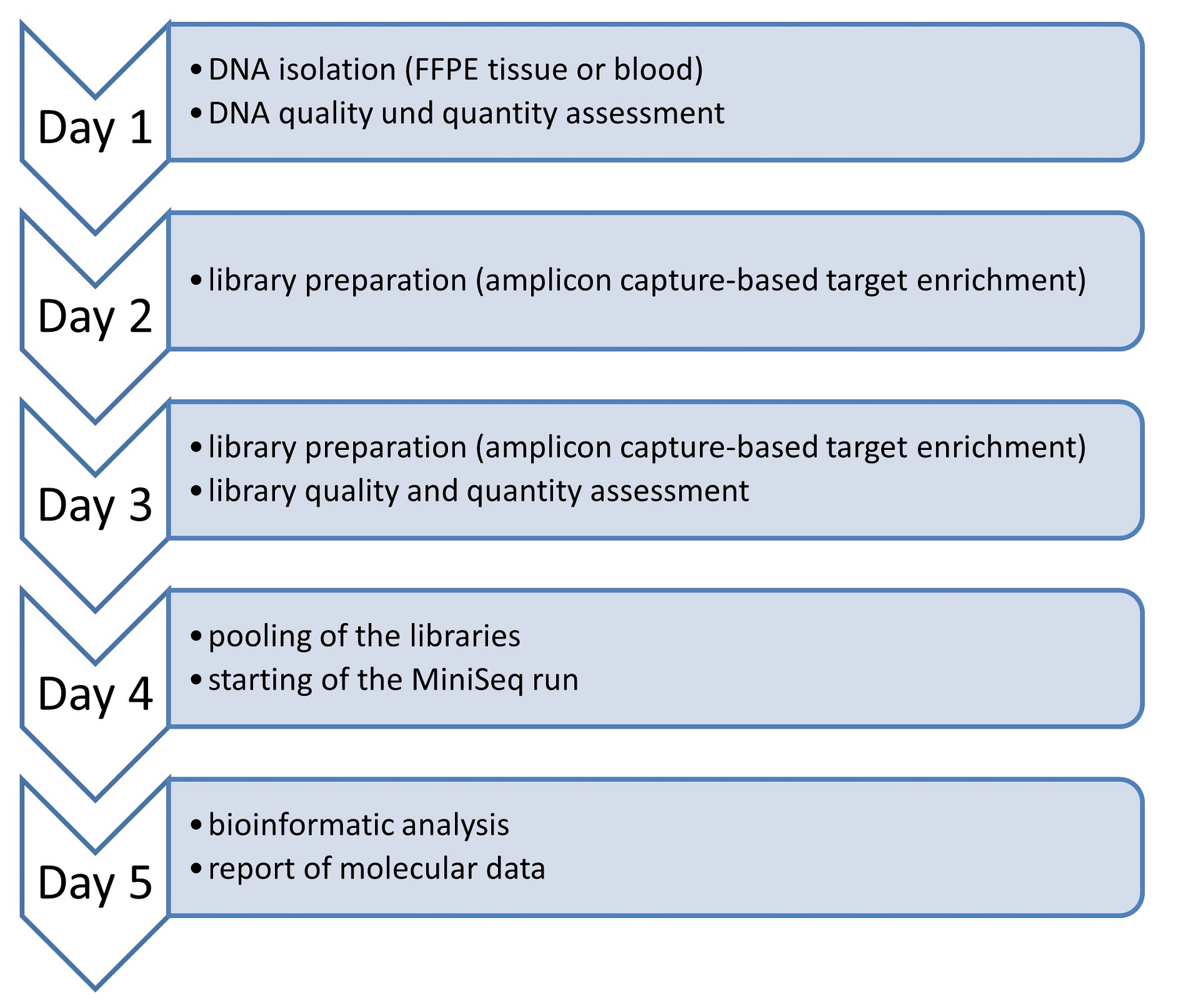

Supplement: Supplementary file 5 — Additional file 5: Supplementary Figure 1. 5-day DNA panel sequencing workflow. [file 40478_2020_1000_MOESM5_ESM.jpg]

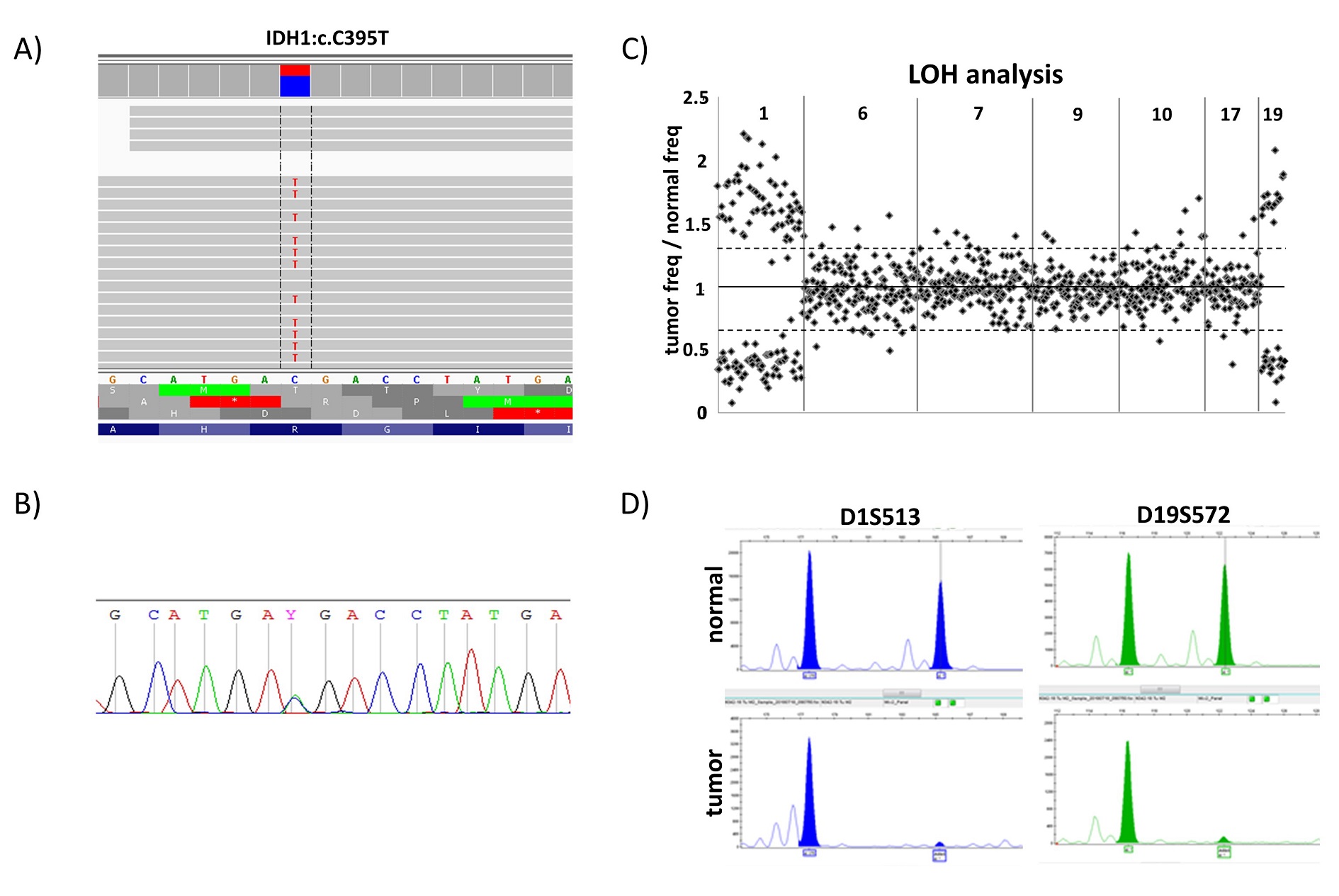

Supplement: Supplementary file 6 — Additional file 6: Supplementary Figure 2. Validation example for an anaplastic oligodendroglioma, IDH-mutant and 1p/19q-codeleted (aO_1). DNA panel result of the IDH1:p.R132H mutation visualized in IGV (A) and corresponding direct sanger sequencing (B). DNA panel results of LOH analysis using commonly occurring SNPs compared to matched blood (C). Corresponding LOH analysis using microsatellite analysis with fluorescence marked oligonucleotides compared to matched blood (D). The results for one marker on chromosomal arm 1p (D1S513) and one on 19q (D19S572) are shown exemplarily. IGV: integrative genomic viewer, LOH: loss of heterozygosity. [file 40478_2020_1000_MOESM6_ESM.jpg]

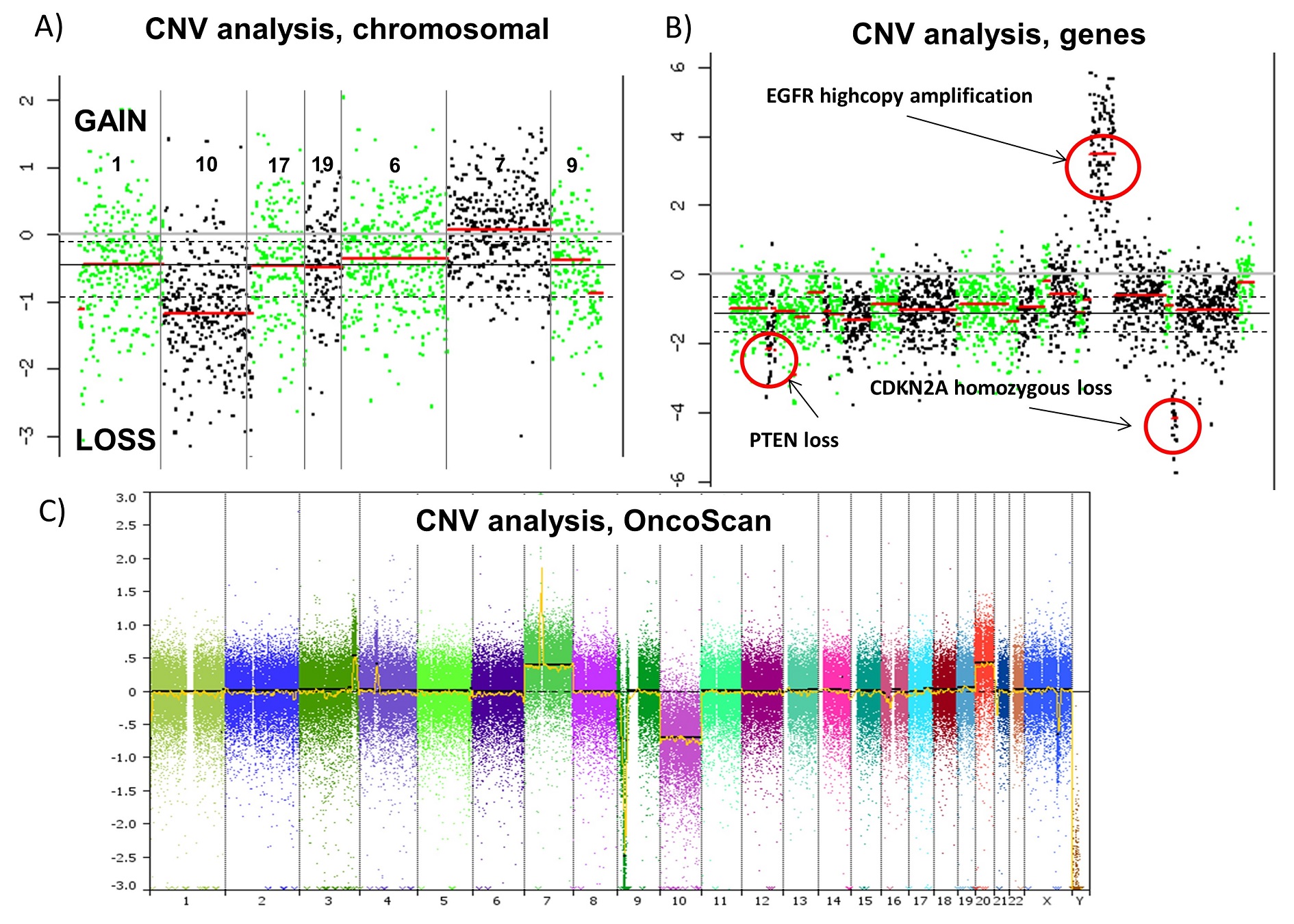

Supplement: Supplementary file 7 — Additional file 7: Supplementary Figure 3. Validation example for the CNV analysis in a case of glioblastoma, IDH-wildtype (GB_17). DNA panel results of the CNV analysis for chromosomal regions (A) and genes (B) were compared to a corresponding OncoScan CNV analysis (C). CNV: Copy number analysis. [file 40478_2020_1000_MOESM7_ESM.jpg]

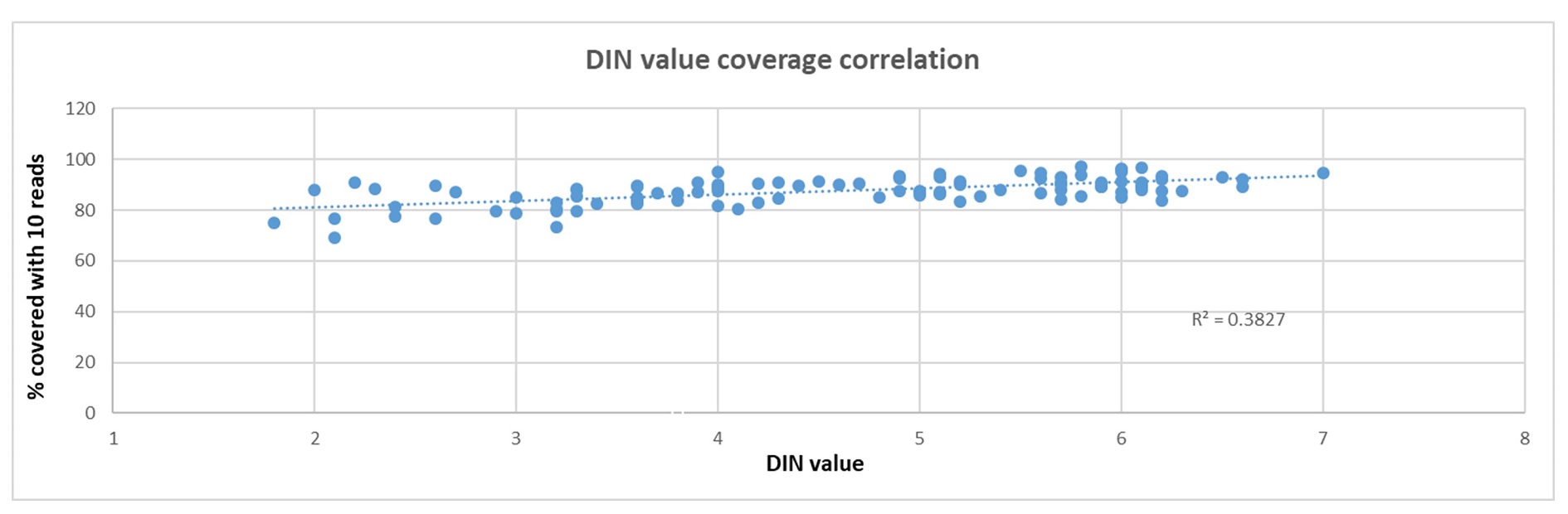

Supplement: Supplementary file 8 — Additional file 8: Supplementary Figure 4. Correlation of coverage with DNA quality. Percentages of target regions covered with at least 10 reads were plotted against the DIN value on a tumor by tumor basis. DIN values were measured with an automated electrophoresis tool (Tapestation 4200, Agilent). Higher DIN values indicate an intact, not degraded DNA and lower DIN values a degraded DNA of low quality. A weak (> 0.3) positive correlation between coverage and DIN value was observed indicating a dependency of coverage on DIN value. [file 40478_2020_1000_MOESM8_ESM.jpg]

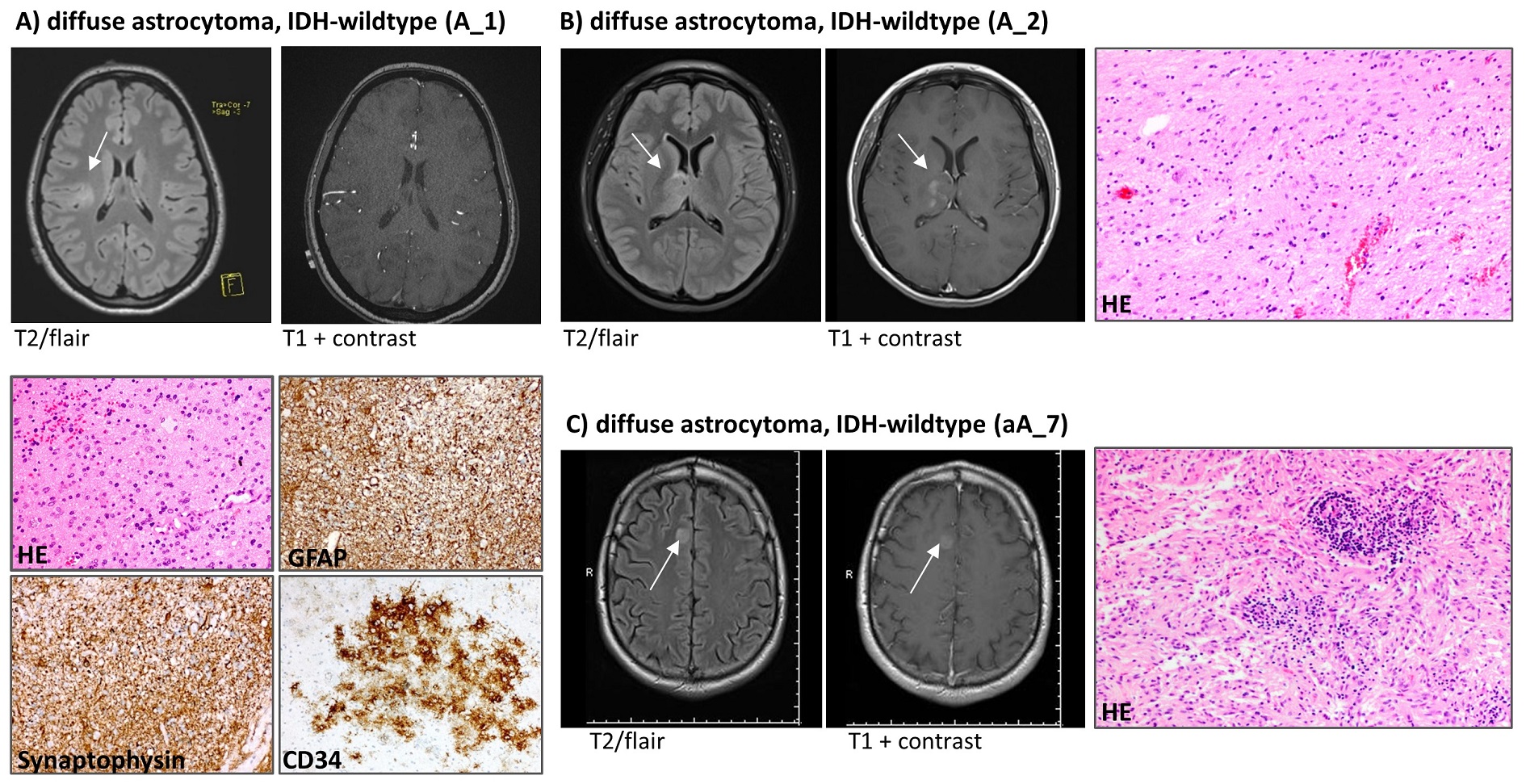

Supplement: Supplementary file 9 — Additional file 9: Supplementary Figure 5. Preoperative magnetic resonance imaging (MRI) and histology of the IDH-wildtype astrocytomas A_1, A_2 and aA_7. (A) A_1. MRI shows hyperintensity in FLAIR without contrast enhancement in T1 in the dorsal region of right insula, compatible with a low-grade glioma. Microscopic examination displayed a neuroepithelial lesion of moderate cell density and only low nuclear pleomorphism. Both GFAP and Synaptophysin were strongly expressed. CD34 immunoreactivity was prominent and highlighted peritumoral satellite cells. (B) A_2. MRI shows several contrast enhancing lesions on both sides of the midline with corresponding FLAIR hyperintensity. Histologically, the lesion exhibited features of a low-grade glioma in a slightly fibrillary background. Cellularity was moderate and mitoses or nuclear atypia of higher degree were absent. Singular entrapped neuronal cells were considered to be pre-existing. (C) aA-7. MRI shows a region of corresponding hyperintensity (FLAIR) and contrast enhancement (T1). Histology revealed a highly cellular astrocytic glioma with moderate pleomorphism, variable cell morphology, sometimes fusiform cells and marked aggregation of lymphocytic infiltrates. [file 40478_2020_1000_MOESM9_ESM.jpg]
